# How AI responds to common HIV/AIDS questions: ChatGPT versus DeepSeek

**DOI:** 10.3389/fpubh.2026.1886493

**Published:** 2026-08-13

**Authors:** Hui Huang, Huichao Zhang, Xinxin Qin, Yuhan Wu, Qi Yu, Mingxia Fang, Binghu Sun, Jian Cheng, Yan Song, Junmei Shu, Ling Wang

**Affiliations:** 1 Department of Nursing, The Second Hospital of Nanjing, Nanjing, China; 2 School of Nursing, Nanjing University of Chinese Medicine, Nanjing, China; 3 Department of Infectious Diseases, The Second Hospital of Nanjing, Nanjing, China; 4 Department of Public Health, The Second Hospital of Nanjing, Nanjing, China

**Keywords:** ChatGPT-3.5, Deepseek-R1, AIDS, HIV, health knowledge

## Abstract

**Background:**

Artificial intelligence (AI) has garnered significant attention and, to some extent, has even replaced certain search engines as a popular channel for people across regions to access information. HIV/AIDS is a common topic among infectious diseases, and patients or high-risk groups frequently search for information online. Our study evaluated the accuracy of two AI models (ChatGPT-3.5 and DeepSeek-R1) in answering HIV/AIDS-related questions.

**Methods:**

This was a cross-sectional analytical study comparing two advanced large language models representing Eastern and Western cultural backgrounds, respectively, with a gold standard (an infectious disease specialist). We compiled 26 common questions related to HIV/AIDS and categorized them into four themes (basic knowledge, diagnosis, treatment, and prevention). Three infectious disease experts independently scored the AI models’ responses on a 4-point scale for each answer. There was no statistically significant difference in the scores for HIV/AIDS-related questions between the two AI models: Z = −2.135, two-sided *p*-value = 0.451 (*p* > 0.05). DeepSeek-R1 demonstrated higher accuracy, with 53.85% of its responses rated as “Excellent,” compared to 48.72% for ChatGPT-3.5; there was no significant difference in accuracy between the two AI models when answering HIV/AIDS-related questions (*χ*^2^ = 0.429, df = 1, *p* = 0.513). Both AI models achieved high average composite scores (DeepSeek-R1 = 3.50, ChatGPT-3.5 = 3.44, out of a maximum of 4 points). The AI models performed exceptionally well across all domains, though they were relatively weaker in the “Treatment” domain.

**Conclusion:**

Our study has identified the potential of AI models, particularly ChatGPT-3.5 and DeepSeek-R1, to provide accurate and comprehensive responses to HIV/AIDS-related questions. As free online tools, they can provide personalized, useful medical information to diverse populations across regions. However, medical information generated by AI models should still be used under the supervision and review of healthcare professionals.

## 1 Introduction

According to data from the 2021 Global Burden of Disease Study, HIV infection remains one of the most serious public health challenges of the 21st century (1). As of the end of 2024, there were still approximately 40.8 million people living with HIV worldwide, with 1.3 million new infections and approximately 630,000 AIDS-related deaths that year (2). As a country with a population exceeding 1.4 billion, China also faces a heavy disease burden. According to data from the Chinese Center for Disease Control and Prevention (3), as of the end of 2024, a total of 1.355 million cases of HIV infection and AIDS had been reported nationwide, with 101,000 new cases that year. While the epidemic remains at a low prevalence level overall, the risk of transmission persists. Compared to their peers, people living with HIV/AIDS have severely compromised immune systems and face potential threats from various opportunistic infections, such as Pneumocystis pneumonia, tuberculosis, and bacterial infections, as well as AIDS-related malignancies (4, 5). For patients, access to accurate information (regarding diagnosis, prevention, and treatment) is key to effectively managing the disease. In the past, patients primarily obtained treatment information from their attending physicians. With the rise of the Internet, however, it has become one of the main channels through which patients access HIV/AIDS-related information, owing to its abundance of resources and ease of use (6). However, the sheer volume of online information and its varying quality have led to low levels of trust in the information patients find (7). Therefore, ensuring that people living with HIV/AIDS can access timely, efficient, and accurate online information is an urgent issue that needs to be addressed.

Large language models are deep learning models based on multi-layer neural network architectures. They are trained in an unsupervised manner on vast amounts of web pages, books, and academic literature, and continuously optimize their performance as they accumulate experience, aiming to simulate intelligent human behavior through algorithms and systems (8). In recent years, artificial intelligence technologies centered on deep learning have achieved significant breakthroughs in image processing, clinical decision support, and the development of personalized treatment plans (9–11). Several studies (12–14) have reported on the application of AI in global HIV/AIDS care. These studies include the use of AI and machine learning models for prediction and risk identification; an exploration of AI’s role in HIV/AIDS management and treatment outcomes; and the use of AI models to provide counseling and advice to people living with HIV.

However, previous studies have the following limitations: First, most previous studies used premium versions that require a paid subscription, which do not reflect real-world user behavior. In practice, most users prefer the free version and ask questions directly. Second, existing research has primarily focused on mainstream international models, lacking a systematic evaluation of the Chinese context and models developed in China. Therefore, this study simulates real-world user behavior to systematically evaluate the accuracy of the free versions of DeepSeek-R1 and ChatGPT-3.5 in answering HIV/AIDS-related questions, and preliminarily investigates whether they have the potential to provide accurate HIV/AIDS-related knowledge.

## 2 Materials and methods

### 2.1 Study design

Our study was conducted from April 8, 2025, to July 15, 2025, at the Department of Infectious Diseases, Nanjing Second Hospital, China. This study involved a cross-sectional analysis of two generative AI language models (ChatGPT-3.5 and DeepSeek-R1) and compared them with a gold standard (an infectious disease specialist). The objective of our study was to simulate real-world user behavior and explore potential applications of AI, particularly the free versions of ChatGPT-3.5 and DeepSeek-R1, for providing HIV-related information to the general public, as well as to evaluate the accuracy of the generated responses against our gold standard (an infectious disease specialist). Responses to these questions were generated using ChatGPT-3.5 and DeepSeek-R1 (by HH).

### 2.2 Data collection

**Figure 1:**
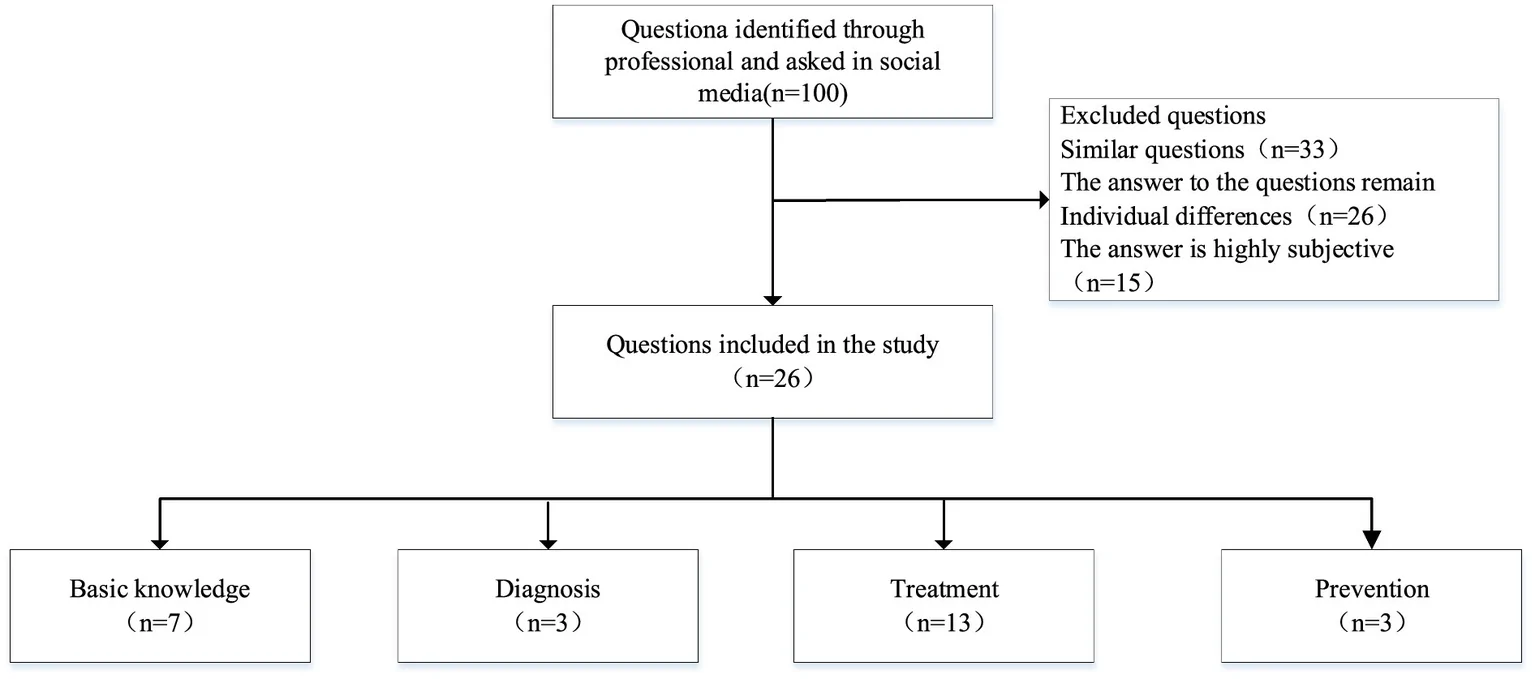
Flowchart of questions selection for HIV/AIDS.

Two nurses specializing in infectious disease research (Hui Huang and HuiChao Zhang), in collaboration with a graduate student in nursing, carefully developed 26 questions related to HIV/AIDS. This extensive information on HIV/AIDS was initially sourced from authoritative online resources, including the Chinese Guidelines for the Diagnosis and Treatment of HIV/AIDS (2024 Edition) (15), well-known publications, the AIDS Association Research Institute, and Dingxiangyuan. Subsequently, the research team revised these questions, selecting those most frequently raised by patients and their primary healthcare providers in clinical settings (Supplementary Table 1). The exclusion criteria were as follows: (1) duplicate questions; (2) questions likely to vary by individual (e.g., “What is my risk of developing cancer after HIV infection?”); (3) questions without clear answers or that are highly subjective (e.g., “What should I do if I’m very anxious after being diagnosed with HIV?”). To further evaluate the strengths and weaknesses of large language models across various subject areas, all questions were categorized into four dimensions: basic knowledge, diagnosis, treatment, and prevention. The question screening process is shown in Figure 1.

### 2.3 Testing environment

This study selected the free versions of ChatGPT-3.5 and DeepSeek-R1 to simulate real-world user behavior, as the vast majority of the general public accesses AI-generated health information through free channels; therefore, evaluating the free versions is more relevant than evaluating the paid versions.

The tested models were the free web version of ChatGPT-3.5 and the free version of DeepSeek-R1 (version DeepSeek-R1-202501). Both models retained their default settings; online search was not enabled, and parameters such as temperature were not modified; each question was submitted independently in a new session. The test hardware consisted of a Lenovo Xiaoxin Air-14API2019 laptop running Windows 10 with the Microsoft Edge browser (version 135.0.3179.54).

Our study only evaluates the quality of publicly generated text content from ChatGPT-3.5 and DeepSeek-R1. It does not involve human subjects, nor does it collect any personally identifiable information, biological samples, or clinical data from patients. Furthermore, it does not involve any interventions on subjects. The risk is extremely low, and there are no privacy breaches or ethically sensitive issues; therefore, ethical review is not required. Consequently, it is deemed unnecessary to submit to ethics committee review.

Subsequently, from May 2, 2025, to May 8, 2025, the three authors (Huang Hui, Zhang Huichao, and Wu Yuhan) each posed the same questions to ChatGPT-3.5 and DeepSeek-R1 and obtained answers. We used the version of ChatGPT-3.5 that provides answers free of charge and DeepSeek-R1, which offers free basic services to the public, to simulate scenarios in which the general public and patients rely on AI tools to obtain HIV/AIDS-related information. Given that the two most widely used AI tools among Chinese people are ChatGPT-3.5 and WeChat, we utilized two chatbots, DeepSeek-R1 and ChatGPT-3.5, both of which provide information free of charge. When querying ChatGPT-3.5 and DeepSeek-R1, the results were based solely on the final content presented; the AI’s thought process was not considered part of the result. The accuracy of the AI models’ responses was independently evaluated by three experienced infectious disease specialists (Mingxia Fang, Binghu Sun, and Jian Cheng), all of whom have over 10 years of experience treating individuals with HIV infection or AIDS. To ensure the objectivity of the scoring, the names of the chatbots were concealed during the evaluation process, and they were identified as A1 and A2. Each response was scored on a 4-point scale: (a) “excellent” indicates completely correct: 4 points; (b) “good” indicates partially correct (correct but incomplete, with room for supplementation): 3 points; (c) “moderate” indicates misleading (a mix of partially correct and incorrect information): 2 points; (d) “poor” indicates completely incorrect: 1 point.

## 3 Statistical analysis

Statistical analysis was performed using SPSS 23.0. Descriptive analysis of the scores assigned by ChatGPT-3.5 and DeepSeek-R1 to HIV/AIDS-related questions was conducted using percentages. Central tendency and dispersion were described using mean ± standard deviation, while the median and range were also reported to provide a more comprehensive picture of the distribution of scores. We used the Wilcoxon signed-rank test to compare the differences in overall scores between the two AI models across 26 questions. Paired observations were defined as the respective scores of DeepSeek-R1 and ChatGPT-3.5 for the same question; each question formed a pair of scores, resulting in a total of 26 pairs. Since the score data were ordered categorical variables on a 4-point scale and did not meet the assumption of a normal distribution, this nonparametric test was selected. A *p*-value of < 0.05 was considered statistically significant. Reliability was assessed using the intraclass correlation coefficient (ICC) to evaluate the consistency of ratings among the three infectious disease specialists. A *p*-value < 0.05 was considered statistically significant.

## 4 Results

**Figure 2:**
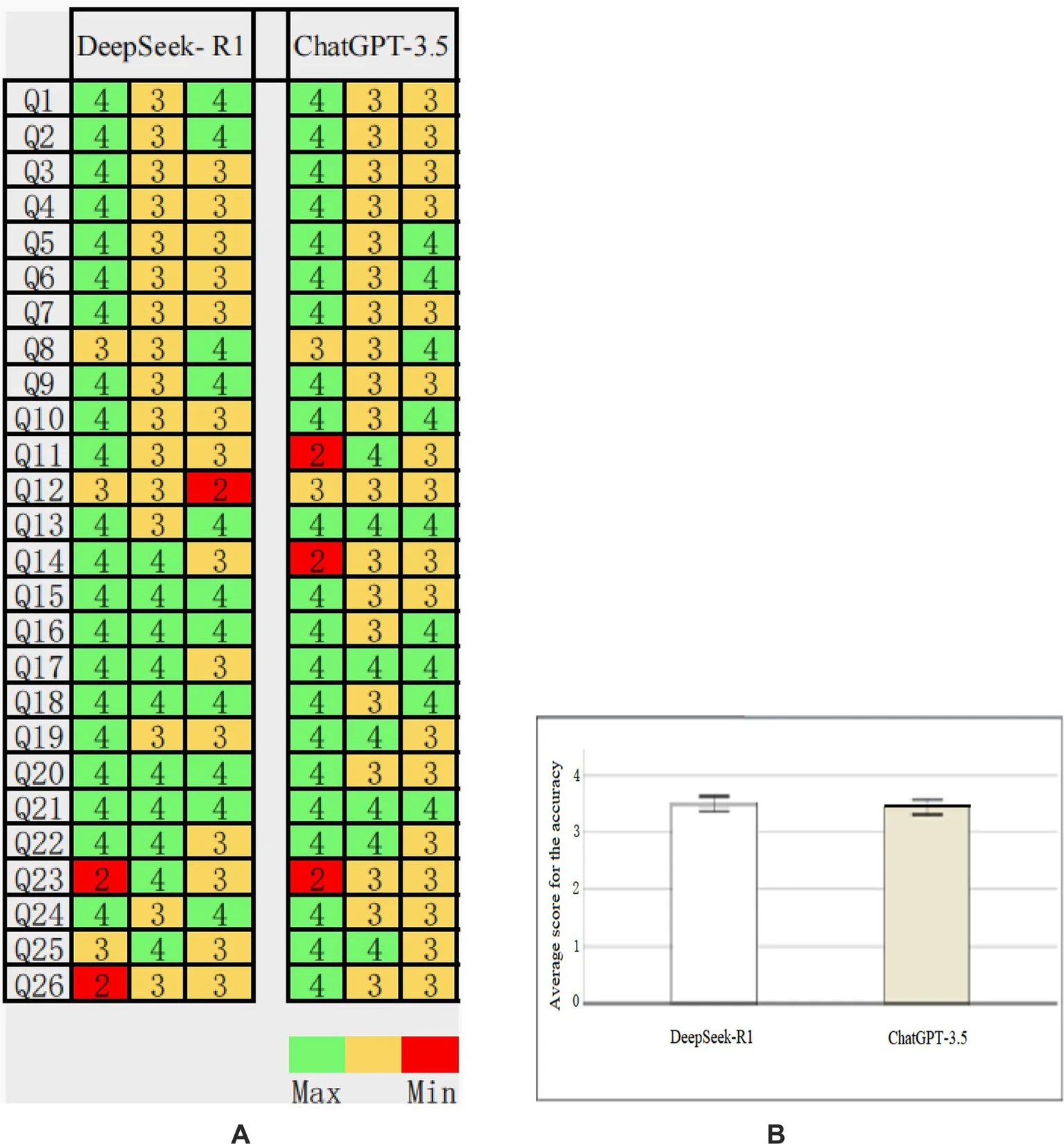
Performance of two AI models in terms of accuracy and comprehensiveness, as evaluated by three infectious disease specialists. **(A)** Individual accuracy scores for each model; **(B)** Mean accuracy scores across all evaluators. AI, Artificial Intelligence.

We collected 100 HIV/AIDS-related questions and ultimately included 26 common questions for analysis. These 26 questions were categorized into the following four groups: basic knowledge (*n* = 7), diagnosis (*n* = 3), treatment (*n* = 13), and prevention (*n* = 3) (Figure 1). Figure 2 details the specific scores assigned by three infectious disease specialists to the 26 standardized HIV-related questions, and Table 1 summarizes the scores of their responses. The average score given by the three infectious disease specialists for DeepSeek-R1’s responses to HIV-related questions was 3.50 ± 0.575, which was higher than that of ChatGPT-3.5 (3.44 ± 0.572). The highest and lowest scores for both DeepSeek-R1 and ChatGPT-3.5 were 4 and 2, respectively; the median score for DeepSeek-R1 was 4, which was higher than ChatGPT-3.5’s median of 3. Z = −2.135, with a two-sided *p*-value of 0.451 (*p* > 0.05), indicating no statistically significant difference.

**Table 1:** Scoring situation and difference in grading between the three reviewers.

AI category	Score			
	Mean±SD	Minimum	Maximum	Median
DeepSeek-R1	3.5 ± 0.575	2	4	4
ChatGPT-3.5	3.44 ± 0.572	2	4	3

**Figure 3:**
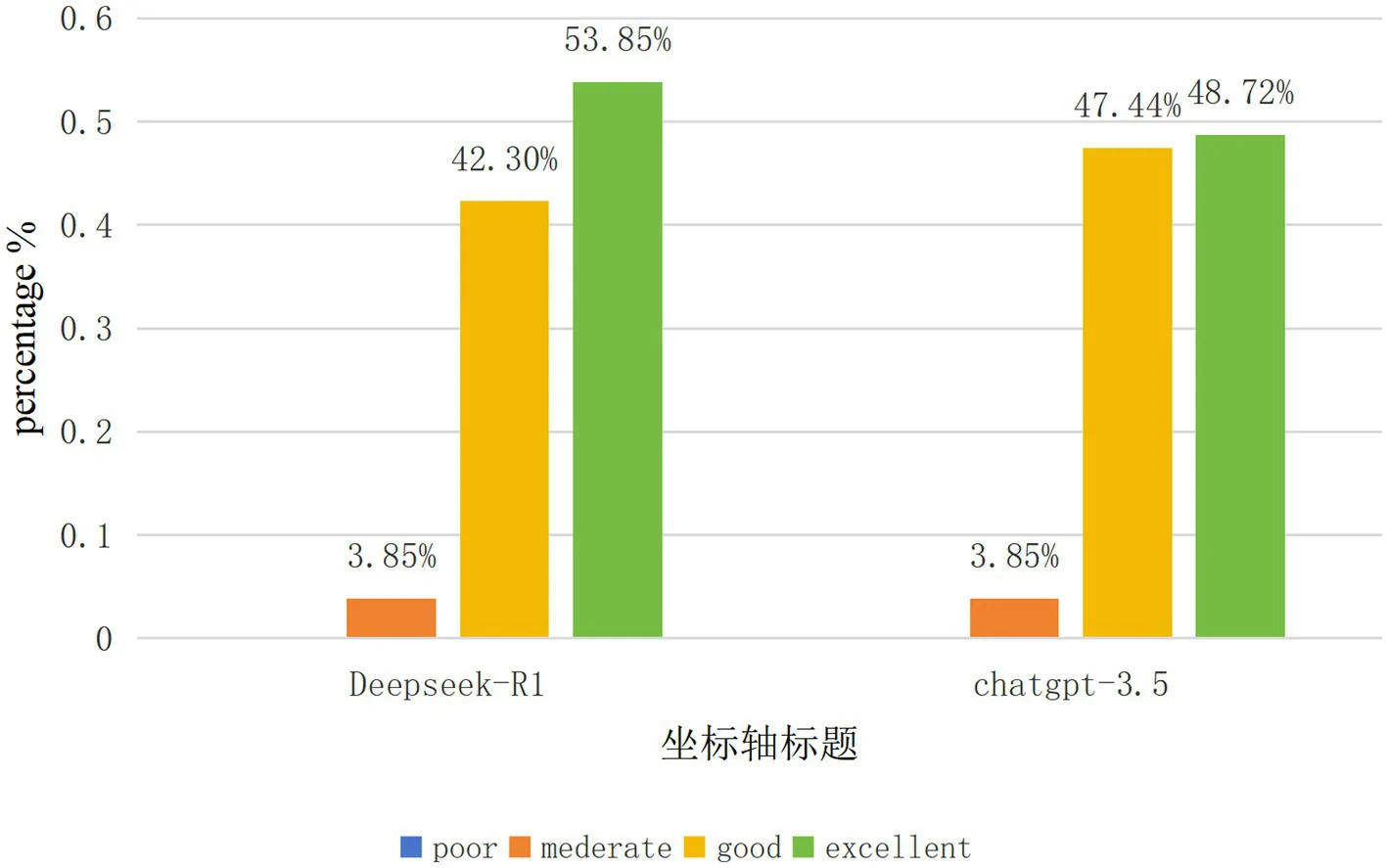
Consensus-based accuracy ratings of LLM-chatbot responses to HIV/AIDS related questions.

Figure 3 illustrates the accuracy rates and scores of the two AI models in answering 26 standardized questions. In DeepSeek-R1, 53.85% of the answers were rated as excellent, higher than ChatGPT-3.5 (48.72%); however, the difference was not statistically significant (*χ*^2^ = 0.429, *p* = 0.513). However, 42.3% of DeepSeek-R1’s responses were rated as “good,” which is lower than ChatGPT-3.5’s (47.44%); this difference was not statistically significant (*p* > 0.05). It is worth noting that the proportion of responses rated as “average” was the same for both AI models (3.85%), and no responses were rated as “poor.” For detailed scoring information on each AI’s responses to each question, see Table 2.

**Table 2:** Categorical statistics for consensus accuracy score of LLM-chatbors to HIV/AIDS related questions.

Domain	No of questions	DeepSeek-R1				ChatGPT-3.5			
		Poor	Mederate	Good	Excellent	Poor	Mederate	Good	Excellent
		(1)	(2)	(3)	(4)	(1)	(2)	(3)	(4)
Basic knowledge	7*3	0 (0)	1 (4.8%)	11 (52.4%)	9 (42.9%)	0 (0)	1 (4.8%)	13 (61.9%)	7 (33.3%)
Diagnosis	3*3	0 (0)	0 (0)	5 (55.6%)	4 (44.4%)	0 (0)	0 (0)	4 (44.4%)	5 (55.6%)
Treatment	13*3	0 (0)	2 (5.1%)	11 (28.2%)	26 (66.7%)	0 (0)	2 (5.1%)	17 (43.6%)	20 (51.3%)
Prevention	3*3	0 (0)	0 (0)	6 (66.7%)	3 (33.3%)	0 (0)	0 (0)	4 (44.4%)	5 (55.6%)

Numbers represent quantities corresponding to categories. Numbers within brackets are percentages (%).

Table 2 presents a comprehensive analysis of accuracy scores for HIV/AIDS-related knowledge, broken down by category. Overall, the two AI models performed consistently across the “Basic Knowledge,” “Diagnosis,” “Treatment,” and “Prevention” categories, with more than 90% of responses rated as “Good” or “Excellent.” Specifically, both DeepSeek-R1 and ChatGPT-3.5 achieved 100% “Good” or higher ratings in the “Diagnosis” and “Prevention” categories. However, DeepSeek-R1 and ChatGPT-3.5 each received a “moderate” rating for one response in the “Basic Knowledge” category and two responses in the “Treatment” category. In particular, the two AI models performed slightly less well in the “Treatment” category, where a higher proportion of responses received “moderate” ratings. It is worth noting that no AI model received a “poor” rating for any of its responses.

Table 3 presents the results of a measurement of the degree of agreement among the three evaluators. The single-measurement ICC for the DeepSeek-R1 group was 0.628 (95% CI: 0.422–0.786), and for the ChatGPT-3.5 group, it was 0.656 (95% CI: 0.457–0.806). Both ICC values fall within the moderate-to-good range, indicating acceptable consistency in the ratings given by the three experts for both models.

**Table 3:** Inter-rater reliability for both models.

Model	ICC(2,1)	95%CI
DeepSeek-R1	0.628	0.422–0.786
ChatGPT-3.5	0.656	0.457–0.806

Two-way random effects model with absolute agreement.

Percentages may not add up to 100% due to rounding.

## 5 Discussion

Although previous studies (12–14) have evaluated the application of AI in the field of HIV/AIDS, this study is unique in terms of model selection (DeepSeek-R1 was trained primarily on Chinese data, while ChatGPT-3.5 was trained primarily on English data) and evaluation conditions (free versions, simulating real-world user behavior). Unlike previous research (16), which primarily focused on assessing the accuracy of ChatGPT, a single large language model, in answering common questions about HIV/AIDS, our study examines real-world scenarios. Influenced by different cultural contexts in the East and West, DeepSeek-R1 is increasingly becoming the primary channel for patients and the public in China to access health information, particularly specialized knowledge. This underscores the need for a systematic evaluation of the accuracy and authenticity of the responses provided by DeepSeek-R1 and ChatGPT-3.5. In summary, our study has far-reaching implications and may pave the way for integrating artificial intelligence into HIV/AIDS medical knowledge across regions.

Our study comprehensively evaluated the ability of DeepSeek-R1 and ChatGPT-3.5 to answer common patient questions. The results indicate that both AI models can provide accurate, comprehensive answers to AIDS-related questions. In tests in which the two AI models answered AIDS-related questions, DeepSeek-R1 outperformed ChatGPT-3.5, achieving a higher average accuracy and a higher proportion of “excellent” ratings (Figures 2, 3), as shown in Table 2. To date, the application of large language models (LLMs) in the healthcare field remains in its early stages, with studies focusing on osteoarthritis (17), *Helicobacter pylori* infection (18), cancer patients (19), and people living with HIV/AIDS (20). However, few studies have compared the performance of DeepSeek-R1 and ChatGPT-3.5 across both Chinese and Western cultures. However, based on research findings from other fields (e.g., hematology) (21), DeepSeek-R1 demonstrates higher accuracy and reliability than ChatGPT-3.5 (DeepSeek-R1: 93% vs. ChatGPT-3.5: 90%). Similarly, U. S. scholars Ibrahim et al. (22) reported that in the American Gastroenterological Association’s self-assessment test, both the base model of DeepSeek (77.1%) and its search-enhanced model (81.5%) outperformed ChatGPT-3 (65.1%) and ChatGPT-4 (62.4%). One potential explanation for DeepSeek-R1’s superior performance is that its training data is relatively more recent. ChatGPT-3.5’s knowledge cutoff is September 2021, which may limit its ability to accurately address current or emerging medical issues (8). In contrast, DeepSeek-R1’s training data is current as of July 2024, which may provide it with more up-to-date medical knowledge. However, since this study did not directly assess the impact of training data recency on model performance, this explanation remains preliminary and requires further research to validate. It should be noted, however, that both models were tested with the online search feature disabled; therefore, the observed performance differences likely reflect differences in their respective pre-training data rather than differences in their ability to retrieve real-time information.

In the four areas of basic knowledge, diagnosis, treatment, and prevention of HIV/AIDS, both AI models achieved satisfactory scores. They consistently advised users to follow their doctors’ recommendations when answering questions related to diagnosis and treatment. This indicates that both AI models can provide patients with accurate and comprehensive information, thereby reducing, to some extent, the risk of treatment failure due to misinformation on the Internet. Although DeepSeek-R1 and ChatGPT-3.5 performed slightly less well when answering questions related to the “treatment” domain (Table 2), their overall performance was good. These results suggest that both can serve as auxiliary tools to help patients acquire knowledge about the disease. However, the transition from information acquisition to actual action is a complex process influenced by multiple factors; therefore, further research is needed to determine whether AI models can improve patients’ medication adherence.

With the widespread use of antiretroviral therapy (ART), AIDS has evolved from an incurable, highly infectious, and extremely lethal disease into a manageable chronic infectious disease (23). However, due to issues with medication adherence, some patients still experience treatment failure (24). Previous studies have shown that higher medication adherence among AIDS patients can increase life expectancy (25, 26). Therefore, improving medication adherence to highly active antiretroviral therapy (HAART) among AIDS patients has become an urgent issue that needs to be addressed. However, it is difficult to achieve satisfactory adherence when patients lack sufficient understanding of HIV/AIDS-related knowledge (27). Various factors, including the long duration of treatment, the high frequency of daily dosing, large drug doses, and medication side effects, further exacerbate this situation. One approach to improving patient adherence is to raise awareness of the disease (28). Previous studies have shown that current methods used to improve patient adherence include adherence-monitoring apps, electronic adherence monitors, digital pill systems, and mobile phone or text message services (29). However, because these methods require significant human and material resources, they are both costly and time-consuming.

In recent years, thanks to its unique advantages, the Internet has attracted approximately 70 to 80% of users to search for health-related information online; however, traditional search engines present a vast amount of information of varying quality, requiring users to expend a great deal of time and effort (30–32). Research has shown (33) that HIV self-management based on patient education is key to controlling the disease, and the effectiveness of such education largely depends on the accuracy and completeness of the information itself. It follows that patients can develop a correct understanding of the disease and consequently adopt effective self-management behaviors only after obtaining reliable information. With advances in technology, artificial intelligence has demonstrated tremendous potential for HIV health education (34). AI models generate concise, intuitive answers to patients’ questions, saving a great deal of time. As online tools, DeepSeek-R1 and ChatGPT-3.5 offer unique advantages, providing users with free access, real-time responses, and personalized content; particularly for patient groups with limited resources, they lower the barrier to accessing specialized knowledge. However, the journey from information accessibility to improved patient health outcomes, including enhanced adherence, narrowed health disparities caused by wealth gaps, and alleviated psychological and disease burdens, remains a complex process involving changes in information-seeking and behavioral patterns (35–37), and further clinical research is needed.

Furthermore, although the results of this study show that both AI models provided accurate and comprehensive responses to AIDS-related questions, it must be acknowledged that certain AI-generated misinformation could endanger patient safety. First, the issue of hallucinations (38): LLMs may generate answers that appear reasonable but are in fact fictitious or incorrect. In this study, although both models performed well on most basic questions, they produced inaccurate or ambiguous information when addressing complex clinical issues such as specific drug dosages and treatment regimens. Second, the risk of spreading misinformation. On the one hand, AI models’ responses are based on their training data, which may contain outdated, incomplete, or biased medical information (39); on the other hand, the models cannot distinguish between high-level evidence sources (such as clinical guidelines and systematic reviews) and low-level evidence sources (such as personal blogs and forum discussions), thereby incorporating unreliable content into their responses (40). Third, there is a potential threat to patient safety. Although the model in this study demonstrated accuracy approaching that of experts in certain areas, it must be emphasized that AI models currently cannot replace the professional judgment of clinicians. In medical decision-making, inaccurate information, incorrect diagnostic suggestions, and treatment recommendations may all lead to delays in patient care, affecting treatment outcomes, causing adverse drug reactions, or even endangering patients’ lives.

## 6 Limitations

This study has several limitations. First, to simulate real-world user behavior, this study used the model’s free version and obtained responses through direct questioning rather than prompt engineering. It should be noted that the free version suffers from issues such as unclear model versions, opaque parameter settings, model updates over time, and response variability (41); these factors all limit the study’s reproducibility and transparency. Second, this study focused on two models, including DeepSeek-R1, a Chinese model, and ChatGPT-3.5, a mainstream international model, to compare model performance across different linguistic and cultural contexts. However, including only these two models for comparison and lacking repeated testing makes it difficult to account for the potential impact of different architectural designs, training data, and parameter scales on model performance, thereby limiting the generalizability of this study’s conclusions. Third, the 26 questions in this study (including 13 treatment-related questions [50%], 7 on basic knowledge, 3 on diagnosis, and 3 on prevention) were selected by the researchers based on internet sources and clinical guidelines. The representativeness of these questions may have been influenced by the researchers’ subjective judgments, resulting in selection bias. This may be because, in simulating real-world user behavior, this study found that in clinical practice, patients pay far more attention to treatment-related questions than to other areas, which, to some extent, reflects the structure of information needs among people living with HIV/AIDS in the real world. However, it must be acknowledged that this imbalance may significantly affect the model’s overall accuracy scores in this domain, thereby limiting the generalizability of the study’s results. Fourth, in this study, DeepSeek-R1 outperformed ChatGPT-3.5, which may be partly attributed to differences in the recency of their training data. ChatGPT-3.5’s training data was current as of September 2021 (42), whereas DeepSeek-R1’s foundation model data was updated through July 2024 (43). Outdated information may affect the AI models’ accuracy in assessing recent or current events; however, because their responses are based on training on various internet texts rather than clinical evidence, inaccuracies in the source material may influence their results.

Therefore, future research should expand the scope of the models (to include Gemini, Claude, DouBao, etc.) and evaluate both free and paid versions separately, while increasing the number of questions and ensuring a reasonable distribution across various fields; adopt a standardized testing environment, such as using API versions; systematically compare different prompting strategies and conduct multi-round repeated testing; establish detailed scoring criteria to improve rater consistency; and validate the results in real-world clinical settings and across a wider range of linguistic and cultural contexts. These improvements will help further validate the findings of this study. Furthermore, we recommend that healthcare professionals regularly review, verify, and supervise medical information generated by AI models. This may help provide patients with accurate information about HIV/AIDS and enhance their ability to self-manage their condition.

## 7 Conclusion

Our study found that both models performed well when answering HIV/AIDS-related questions, with DeepSeekR1 performing slightly better than ChatGPT 3.5. Although the responses from both AI models were inaccurate in certain areas, such as the timing of ART initiation for adults and adolescents, we believe that with continued advances in AI technology, DeepSeek-R1 and ChatGPT-3.5 can become highly valuable supplementary tools for patients and healthcare providers.

## Data Availability

The original contributions presented in the study are included in the article/Supplementary material, further inquiries can be directed to the corresponding author/s.
